# How can simple household procedures reduce exposure to pesticides from fruits and vegetables: current habits and recommendations

**DOI:** 10.2478/aiht-2025-76-3984

**Published:** 2025-06-30

**Authors:** Antonija Sulimanec, Jelena Kovačić, Marija Macan, Martina Pavlić, Irena Keser, Veda Marija Varnai

**Affiliations:** Institute for Medical Research and Occupational Health, Division of Occupational and Environmental Health, Zagreb, Croatia; Croatian Agency for Agriculture and Food, Centre for Food Safety, Osijek, Croatia; University of Zagreb Faculty of Food Technology and Biotechnology, Zagreb, Croatia

**Keywords:** eating habits, peeling, pesticide residues, washing food, guljenje, ostatci pesticida, pranje hrane, prehrambene navike

## Abstract

Healthy as they may be, fruits and vegetables may significantly contribute to dietary pesticide intake in modern households. However, certain simple procedures, such as washing and peeling food, can help reduce this intake. Our study looks deeper into the habits of cleaning fruits and vegetables before consumption or cooking in the households of the Croatian capital Zagreb and its surroundings, based on data collected in the first, 2022–2023 wave of a larger cohort study “Exposure to pyrethroid and organophosphate insecticides in children – risk assessment for adverse effects on neuropsychological development and hormonal status”. Data were collected with a questionnaire completed by volunteering parents or legal guardians. Although almost all households reported washing fruits and vegetables before consumption or cooking, over 60 % did not peel fruits and vegetables that can be consumed with the peel, such as apples, pears, peaches, nectarines, and tomatoes (raw and cooked). In addition, we provide general recommendations for reducing dietary exposure to pesticide residues through simple household procedures. Croatian consumers should be better informed about which food products may contribute to higher pesticide exposure and how to reduce it.

## Abstract (hr)

Koliko god zdravo bilo, voće i povrće mogu značajno doprinijeti unosu pesticida u modernim kućanstvima. Međutim, u smanjenju tog unosa mogu pomoći određeni jednostavni postupci, poput pranja i guljenja hrane. Naše istraživanje detaljnije proučava navike čišćenja voća i povrća prije konzumacije ili kuhanja u kućanstvima u Gradu Zagrebu i okolici na temelju podataka prikupljenih u prvom valu kohortnog istraživanja *Izloženost piretroidnim i organofosfatnim insekticidima u djece – procjena rizika od štetnih učinaka na neuropsihološki razvoj i hormonski status*, koji se provodio od 2022. do 2023. Podatci su prikupljeni upitnikom koji su ispunili volonteri roditelji/ili zakonski skrbnici. Iako su gotovo u svim kućanstvima izjavili da peru voće i povrće prije konzumacije ili kuhanja, više od njih 60 % nije gulilo voće i povrće koje se može konzumirati s korom, poput jabuka, krušaka, breskvi, nektarina i rajčica (sirovih i kuhanih). Dodatno, dajemo općenite preporuke za smanjenje izloženosti ostatcima pesticida u prehrani jednostavnim kućanskim postupcima. Hrvatski potrošači trebali bi biti bolje informirani o tome koji prehrambeni proizvodi mogu pridonijeti većoj izloženosti pesticidima i kako je smanjiti.

More than half of the EU population consumes fruit and vegetables at least once a day, and women consume more than men (1). In 2019, the reported daily consumption in the EU was 365 g per capita (2), which is slightly below the recommendations of 400–600 g per day (3).

Besides their nutritional benefits for health, fruits and vegetables are important dietary contributors to pesticide residue exposure. To ensure food safety, the European Commission has issued maximum residue levels (MRLs) in more than 350 foods (4, 5). Every year, over 600 different pesticides are monitored in more than 75,000 food samples, and the European Food Safety Authority (EFSA) issues annual reports on findings and assessments of acute and chronic risks to consumer health. Yet, despite continuous regulatory assessments indicating that dietary exposure to pesticides is unlikely to pose a risk for EU consumers (6), public concern regarding pesticide use and its impact on human health remains high, especially in view of epidemiological reports suggesting that even low pesticide exposure poses a risk to human health, especially in children (7,8,9,10,11,12,13,14,15,16,17,18,19,20,21,22). According to Koch et al. (23), consumers view pesticides as a threat to food safety and most are convinced that pesticide residues in foods are generally not permitted.

Due to these concerns, a large cohort study was launched in Croatia in 2020, aiming to investigate the potential adverse effects of pesticides on children's pubertal and neuropsychological development, “Exposure to pyrethroid and organophosphate insecticides in children – risk assessment for adverse effects on neuropsychological development and hormonal status” (PyrOPECh; HrZZ IP-2019-04-7193), which included (pre)pubertal boys from Zagreb, Croatia and its surroundings (24). The first wave provided data for us to look deeper into the local household fruit and vegetable intake, and habits of cleaning them before consumption or cooking to identify possible issues and to recommend practical steps to reduce dietary exposure to pesticide residues.

## PARTICIPANTS AND METHODS

We relied on preliminary data from the PyrOPECh cohort study on fruit and vegetable consumption and on household methods for cleaning fruits and vegetables before consumption or cooking, collected in 2022–2023. The cohort consisted of 459 pairs of parents/guardians and their sons (aged 11 on average) attending elementary schools in the City of Zagreb and Zagreb County, Croatia. A detailed description of recruitment, rationale for selecting only boys, response rate, and study methodology is provided in the manuscript under review (24). The design and protocol of the PyrOPECh study are briefly described in several conference abstracts (25, 26) and on the PyrOPECh official website (https://pyropech.imi.hr).

Data relevant to this paper were collected through a self-administered food propensity questionnaire (FPQ) completed by the parents/guardians of participating boys. The FPQ was specifically designed to collect data on usual long-term food and beverage intake over the previous year, considering the studied population's local foods and eating habits. Additionally, participants were asked to specify how often they washed fruit and vegetables by selecting between “sometimes/never” and “always/almost always” and what they used to wash them by selecting between water alone, a combination of water and detergent, vinegar, lemon juice, salt water, and baking soda. Parents/guardians were also asked to report whether the child ate certain fruits and vegetables (apple, aubergine, baked potato, cooked cucumber, cooked tomato, nectarine, pumpkin, peach, pear, zucchini) with or without peel.

The PyrOPECh study, from which we draw preliminary data, was designed and conducted in accordance with the Declaration of Helsinki (2013, 2024) and was approved by the Ministry of Science and Education and Youth (Class: 602-01/21-01/00298, Reg. No.: 533-05-21-0004), school principals, and the ethics committees of the Institute for Medical Research and Occupational Health (IMROH) (Reg. No.100-21/19-14) and collaborating institutions. Participating boys and their parents/guardians signed informed consents to providing biological samples and to the use of all analytical results and personal data. Parents/guardians were involved as a proxy to assist their children in recording food intake.

## RESULTS AND DISCUSSION

The results of our preliminary study show that almost all participating Zagreb households wash fruits and vegetables before consumption or cooking, most often with water, which is in line with the general recommendations issued by food safety authorities worldwide (27,28,29,30).

**Figure 1:**
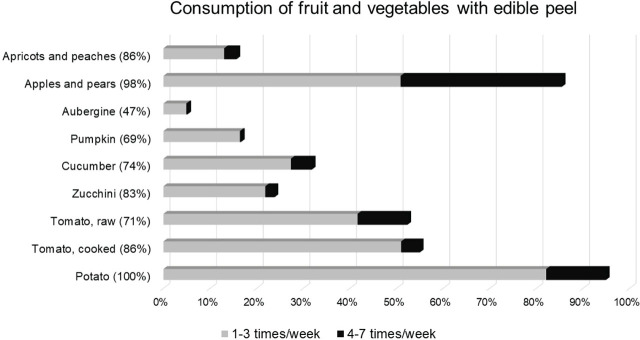
Consumption frequency of certain fruits and vegetables with edible peel in the 1^st^ (2022–2023) wave of the PyrOPECh cohort study (N=459). For each food item, the percentage of consumers in the whole sample is shown

Figure 1 shows the frequency of consumption of certain fruits and vegetables in the PyrOPECh cohort. Apples/pears, apricots/peaches, tomatoes (raw or cooked), and potatoes were consumed by over 85 % of participants. Apples/pears were consumed often (4–7 times/week) by 35 % of respondents. Raw tomatoes and cucumbers were consumed less often (1–3 times/week) by 42 % and 27 % of participants, respectively. Most participants (82 %) reported to consume potatoes 1–3 times/week. Only 5 % ate aubergines at least once a week.

**Figure 2:**
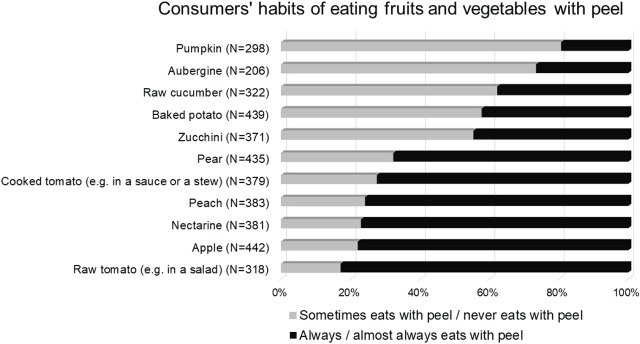
Consumers' habits of eating fruits and vegetables with peel in the 1^st^ (2022–2023) wave of the PyrOPECh cohort study. The percentage is expressed only for those participants who stated that their son eats a respective food item (the number of those participants is shown in brackets). The participants who did not answer the question regarding peeling were excluded from the figure

Figure 2 shows the proportion of participants who consumed peeled fruits and vegetables. More than two thirds reported that they always or almost always ate whole apples, cooked and raw tomatoes, pears, nectarines, and peaches. More than a third reported that they always or almost always ate whole vegetables, such as raw cucumbers and zucchini, and 42 % ate baked potatoes with their peel. In contrast, over 80 % reported that they usually peeled pumpkins, and 73 % that they peeled aubergines.

**Figure 3:**
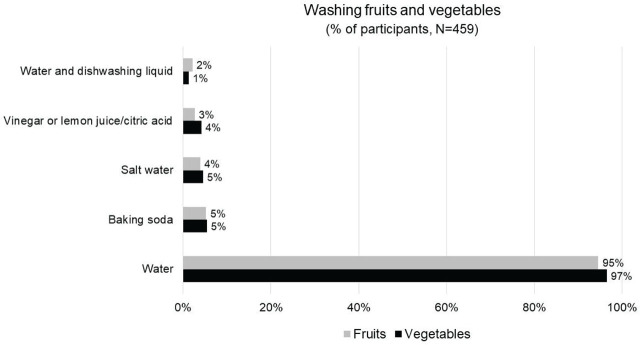
Household methods used for washing fruits and vegetables before consumption in the 1^st^ (2022 – 2023 ) wave of the PyrOPECh cohort study. The percentages refer to the participants who stated that they always or almost always apply a specific cleaning method

Figure 3 shows the distribution of household methods used for washing fruits and vegetables. Most participants (97 %) used water alone, while less than 6 % used any of the other methods such as combining water and dishwashing liquid, vinegar or lemon juice, salt water, or baking soda.

According to the meta-analysis by Keikotlhaile et al. (31), the average pesticide reduction rate with washing and peeling is 76 % and 44 %, respectively. The US National Pesticide Information Center (NPIC) (28) recommends keeping food under running water instead of soaking it. In contrast, there are reports that soaking vegetables in acid, alkaline solutions, or salt can reduce pesticides more effectively than water (31), but few consumers use these to clean food before use, which is also the case in our study cohort (Figure 3). Soaps, detergents, or any commercial produce for washing food are not recommended. According to the United States Food and Drug Administration (US FDA) (29), these ingredients can be absorbed by the fruits and vegetables through skin and may cause gastrointestinal problems.

Considering that most pesticides are applied directly and do not penetrate deep into the crops (27), peeling off the skin or outer layers and leaves is the most effective way to reduce pesticide residues. Even though MRLs are exceeded in less than 1 % of analysed samples in the EU (6), it is recommended to peel fruits before use whenever possible (27). For leafy vegetables like lettuce, the outer layers should be taken off and the remaining produce washed according to recommendations (27, 28).

However, no preparation method can completely remove pesticide residues from food. The reduction rate depends on various factors, including pesticide location in food and their physicochemical properties such as solubility, octanol-water partition coefficient, hydrolytic rate constants, and volatility (33). It is, therefore, recommended to consume a variety of foods from different sources. This reduces the likelihood of exposure to a single pesticide and provides a better mix of nutrients.

On the other hand, washing and peeling may also reduce the nutritional value of fruits and vegetables by removing vitamins, trace elements, dietary fibres and phytochemicals (i.e., quercetin, carotenoids, and anthocyanins) contained in the skin. Therefore, only certain fruits and vegetables that are recognised as potential sources of multiple residues like apples, pears, cucumbers, and zucchini (6) should be peeled.

Organic food should be washed and peeled before consumption as well. Consumers usually purchase organic food to avoid dietary pesticide exposure, but even though they should be produced without the use of synthetic chemicals, organic products are not completely pesticide-free. They may contain very low levels of pesticides, either natural (organic) or can be contaminated indirectly, through their environment. According to Schleiffer and Speiser (34), two monitoring studies recently revealed that 9–28 % of organic products in Switzerland and Germany contained pesticides and attributed their findings to unauthorised pesticide application, false marketing, cross-contamination in transport or food storage, or unintentional contamination via soil, air, water, or rain.

### Study limitations

Speaking about organic food, our findings are limited inasmuch as the questionnaire used in the PyrOPECh study did not differentiate between organic and farm-grown fruits and vegetables, nor could it establish the quality of household preparation procedures. Moreover, like with most questionnaires, self-reporting is prone to some kind of response bias (especially if parents/guardians respond on behalf of their children), and most importantly, the data we have presented here are preliminary and narrow in scope. As the PyrOPECh study continues, we hope that it will shed more light on the associations between pesticide exposure and hormonal development in boys.

## CONCLUSION AND RECOMMENDATIONS

For now, however, our preliminary findings reveal fruit and vegetable consumption and household preparation habits and potential room for improvement in these habits. Croatian consumers should be better informed about which food products may increase pesticide exposure and how food should be prepared before consumption to reduce it. With that in mind, we would like to summarise general recommendations for reducing dietary exposure to pesticides from fruits and vegetables as follows:

- All fresh fruits and vegetables, including organically produced ones, should be washed thoroughly under running tap water;
- Soaps and detergents should not be used to clean food;
- Commercial food preparation detergents are not recommended, as their safety and potentially adverse health effects are still unknown;
- Fruits and vegetables that may contain residues of multiple pesticides should be peeled or the outer layers of leafy vegetables taken off and the rest washed thoroughly;
- Even though many factors may influence the reduction rate of pesticides, consumers should be aware that no preparation method can completely remove pesticide residues;
- Eating a variety of fruits and vegetables from different sources may reduce the likelihood of exposure to a single pesticide.
